# Iatrogenic Kaposi's sarcoma caused by corticosteroids*

**DOI:** 10.1590/abd1806-4841.20165772

**Published:** 2016

**Authors:** Rosana Lazzarini, Andressa Sato de Aquino Lopes, Rute Facchini Lellis, Fabiana Brasil

**Affiliations:** 1 Irmandade da Santa Casa de Misericórdia de São Paulo – São Paulo (SP), Brazil

To the Editor,

Kaposi´s sarcoma (KS) is a malignant neoplasm of endothelial cells with low proliferation, described by Moritz Kaposi in 1872. It is classified into four groups: endemic, reported in African regions; epidemic, which is acquired immune deficiency syndrome(AIDS)-related; classic, at first reported primarily in Ashkenazi Jews and Mediterranean peoples, indicating a genetic relationship; and iatrogenic, which affects immunosuppressed patients of autoimmune and malignant diseases, and transplanted recipients.

**Figure 1:**
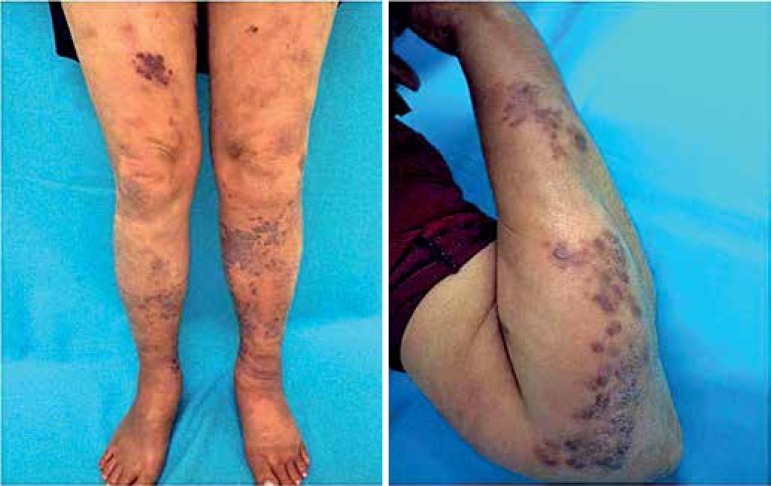
Violaceous and erythematous papules and plaques with irregular and well-defined edges, and smooth and bright surface on the upper and lower limbs

A 36-year-old female patient was diagnosed with lepromatous leprosy in 2008. After a five-month multibacillary treatment (dapsone, rifampicin and clofazimine), erythematous and painful nodules disseminated throughout her body. Type 2 reaction was diagnosed and treated with prednisone 40 mg/day, ranging from 20-60 mg/day according to the patient's response. Treatment lasted between 2008 and 2013 due to persistent lesions. However, a new exam in 2013 showed violaceous and erythematous nodules and plaques, ranging from 1.0-10cm, distributed on the upper and lower limbs, pubis, and left breast. Non-depressible edema was also found on the lower limbs (Figure 1). No lesions were found on oral and genital mucosae.

**Figure 2:**
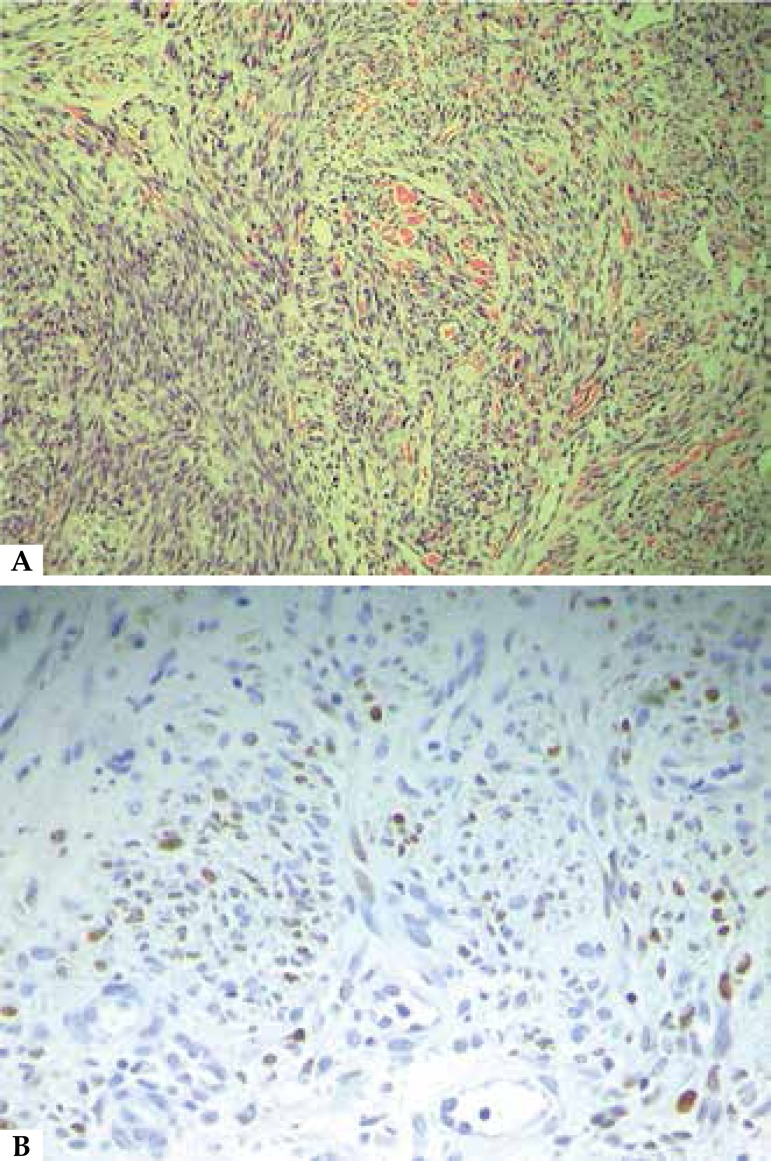
A: Proliferation of fusiform cells, band-forming cells with nuclear atypia and mitotic activity, and extravasation of lymphocytes in the dermis (40x H&E). **B**: Immunohistochemistry positive for human herpesvirus-8 (HHV-8)

The lesion was asymptomatic, and the patient showed no improvement, despite the continued use of high doses of prednisone. SK was considered and confirmed after an anatomopathological examination (Figure 2A). Immunohistochemistry for human herpesvirus-8 (HHV-8) was positive (Figure 2B).

Blood count, liver and kidney exams, serological tests for syphilis, hepatitis B and C and HIV, chest X-ray, and abdominal ultrasound were normal or non-reactive.

The lesion was diagnosed as iatrogenic KS induced by prolonged use of corticosteroids (CS).

**Figure 3:**
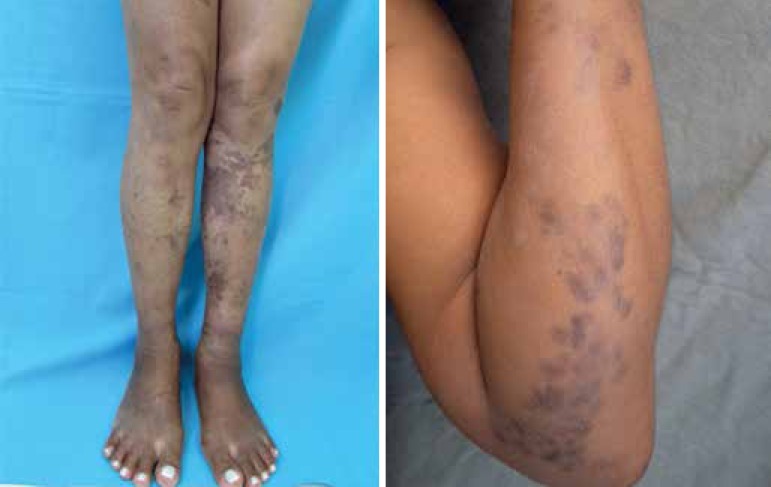
Residual brownish macules on the upper and lower limbs

As a critical therapeutic measure, due to the large number of lesions on different parts of the body, the patient had to stop taking the inducing medication. CS dose was gradually reduced, but the patient showed new erythematous and painful nodules on her upper and lower limbs, accompanied by malaise. It was interpreted as type 2 reaction; clofazimine 300 mg/day was prescribed for 30 days to control the reaction, with gradual dose reduction and the previous slow withdrawal of CS. After three months, the lesions of type 2 reaction disappeared. The medication was fully withdrawn; six months after the KS diagnosis, these lesions improved with residual brownish stain (Figure 3). A three-year follow-up showed persisting residual lesions.

KS in immunosuppressed patients was first reported in transplant recipients under cyclosporine. However, it has also been reported in other haematological, rheumatological, and pulmonary diseases caused by immunosuppressive treatment.^1^

This CS-related disease was found in the treatment of different diseases, such as idiopathic thrombocytopenic purpura, temporal arteritis, Sjogren's syndrome, pemphigus vulgaris, bullous pemphigoid, dermatomyositis, systemic lupus erythematosus, primary biliary cirrhosis, rheumatoid arthritis, and lymphoproliferative disorders.^1,2^ This case reports a patient under treatment of leprosy type 2 reaction. The patient's medical records failed to reveal the time between the beginning of the CS treatment and the emergence of the first KS lesions. In the reviewed literature, the KS may appear between one month and 20 years after the immunosuppressive drug is introduced.^2^

The presence of HHV-8 was first associated with the KS in 1994. The herpesvirus DNA sequence was identified in KS fragments from patients with AIDS^3^ and has subsequently been reported in all KS types. It infects the endothelial cells and remains there in a latent state. When reactivated, it causes aberrant angiogenesis, which is an increase in production and secretion of pro-angiogenic factors.^4^

KS clinical lesions may vary from brownish macules and plaques to violaceous exophytic tumors. Some lesions may ulcerate or invade surrounding tissues, such as the bones. Different stages may coexist, as seen in this case report, but adjacent tissues and inner organs remain intact.^5^

The severe lesions and the different body regions affected required the withdrawal of the immunosuppressive drug prescribed in the earlier therapy. Such measured has been reported by other authors and was successfully reproduced in this case.^2^

KS is a HHV-8-associated tumor and must be taken into account for all cases of patients undergoing immunosuppresseve therapy, regardless of the drug used. As far as it could be assessed, this is the first case report of KS in immunosuppressed patients by CS during leprosy reaction treatment.
